# Psychiatric admission in female survivors of childhood and young adult cancer: a whole population retrospective study

**DOI:** 10.1136/bmjment-2025-301683

**Published:** 2026-02-26

**Authors:** W Hamish B Wallace, Tom W Kelsey, David S Morrison, Katie FM Marwick, Richard Anderson

**Affiliations:** 1 Royal Hospital for Children and Young People, Edinburgh, UK; 2 Computer Science, University of St Andrews, St Andrews, UK; 3 Scottish Cancer Registry, Public Health Scotland, Edinburgh, UK; 4 Division of Psychiatry, The University of Edinburgh College of Medicine and Veterinary Medicine, Edinburgh, UK; 5 MRC Centre for Reproductive Health, University of Edinburgh, Edinburgh, UK

**Keywords:** PSYCHIATRY, Data Interpretation, Statistical

## Abstract

**Background:**

The last 40 years have seen a substantial improvement in overall survival from cancer in children and young people. There is limited information on psychiatric wellbeing in female survivors of cancer at a young age.

**Objective:**

In this 40-year population-based linkage study, we set out to determine the incidence of first psychiatric admission compared with a non-exposed age-matched control.

**Methods:**

Scottish cancer registry records from 1981 to 2012 were linked to psychiatric admissions, maternity and death records from January 1981 to September 2018 using the unique personal Community Health Index number allocated to each person in Scotland. For each exposed subject, three age and deprivation matched controls from the population were selected. The primary exposed group was all females with a cancer diagnosis at age <25 years and no previous pregnancy and no psychiatric admission before cancer diagnosis. The main outcome measure is admission to a psychiatric hospital with a mental health diagnosis.

**Findings:**

Female cancer survivors had a significantly lower cumulative incidence of first psychiatric admissions than matched controls over the 38 years of follow-up for the cohort (p<0.05). The relative risk of a first psychiatric admission at 25 years from cancer diagnosis was 0.72 (0.56–0.89).

**Conclusion:**

Overall, we have shown that young cancer survivors are less likely than age-matched controls to have a psychiatric admission after cancer diagnosis. In particular, psychiatric admissions for mood disorders, neuroses, personality disorders and substance use are significantly less likely in the cancer survivors.

**Clinical implications:**

The experience of cancer treatment and survival in young females may reduce the risk of psychiatric admission in later life.

**WHAT IS ALREADY KNOWN ON THIS TOPIC**

- With increasing overall survival for children and young people with cancer over the last 40 years, the long-term health burden of their diagnosis and treatment is increasingly recognised. There is limited information on psychiatric well-being in female survivors of cancer at a young age with some studies suggesting increased psychiatric morbidity.

**WHAT THIS STUDY ADDS**

- In this whole population Scottish linkage study, we have shown that young female cancer survivors are less likely to have a psychiatric admission after their cancer diagnosis than matched controls.
- Admissions for mood disorders, anxiety and stress-related disorders, personality disorders and substance abuse are significantly less likely in the cancer survivors.
- We have also shown that cancer survivors who have a live birth are less likely to have a psychiatric admission than controls, whose risk of psychiatric admission was increased by childbirth.

**HOW THIS STUDY MIGHT AFFECT RESEARCH, PRACTICE OR POLICY**

- The reasons that young female cancer survivors are less likely to have a psychiatric admission than age-matched controls are unclear but they may be more able to access support and interventions that may avoid psychiatric admission. It is also possible that female cancer survivors, particularly those without a central nervous system diagnosis, are more resilient to severe mental health disorders than non-exposed individuals for reasons which are unclear and require further research.

## Introduction

 There has been a steady improvement in the survival of children and young people with cancer over the last 40 years. Around 80% of people diagnosed with cancer in childhood at ages 15–24 years in the UK survive for 10 years or more.^1^ By 2001,^2^ it was clear that increasing numbers of survivors may have medical problems that require ongoing specialist follow-up, and strategies for long-term follow-up and care needed to be developed and evaluated.

In a seminal study in 2006^3^ using data from the US Childhood Cancer survivor study, Oeffinger *et al* reported the cumulative incidence of a chronic health condition in childhood cancer survivors of 73.4% (95% CI 69.0 to 77.9) 30 years after the cancer diagnosis, with a cumulative incidence of 42.4% (95% CI 33.7 to 51.2) for severe, disabling or life-threatening conditions or death. The burden of chronic health conditions in survivors of childhood cancer is substantial and highly variable.^4 5^ There is mixed evidence of the prevalence of severe psychiatric illness in survivors.

A recent Scandinavian study suggested increased psychiatric ill-health in survivors of childhood cancer (<20 years at diagnosis).^6^ The overall rates of incident psychiatric disorders during the study period were 34% higher among childhood cancer survivors than among the matched population (HR 1.34, 95% CI 1.28 to 1.39). They concluded that childhood cancer survivors are at higher long-term risk of psychiatric disorders than their siblings and matched individuals from the general population. A systematic review in 2018 identified many mental health problems experienced by survivors of childhood cancer; however, the exact incidence, prevalence and risk factors for their development remain unclear.^7^

By accessing whole population Scottish cancer registry records from 1981 to 2012 and linking to psychiatric admissions, maternity and death records using the unique personal Community Health Index (CHI) number allocated to each person in Scotland, we set out to determine the incidence of first psychiatric admission in young female cancer survivors compared with a matched control group. In the first instance, we confined our study to female cancer survivors, allowing us to explore the novel interaction with childbirth as an established major risk factor for psychiatric admission in young women.

## Methods

Scottish cancer registry records from 1981 through 2012 were linked to psychiatric admissions, maternity and death records from January 1981 to September 2018 using the unique personal Community Health Index (CHI) number allocated to each person in Scotland on first registration with the national health service.^8^ The primary cancer survivor group was extracted as all females with a cancer diagnosis at age below 25 years and no previous pregnancy and no psychiatric admission before cancer diagnosis. For each cancer survivor, three females who had not been diagnosed with cancer were selected from the CHI database to provide a population-based control group. The CHI database is a register of all National Health Service Scotland patients. Matching was by age at and year of diagnosis, previous pregnancy and mental health history (i.e. no previous pregnancy nor previous mental health admission), and socio-economic status using deprivation index quintiles based on Scottish postal address data.^9^ Any ages at death were recorded, and in the analysis of time to first psychiatric hospital admission after cancer diagnosis, death was treated as a competing event. This was accounted for by excluding mental health admission records for the matched controls after the date of death for the cancer survivor match. Subjects entered the study on the date of diagnosis and left at death, or end of follow-up. Our study complies with the Strobe-Record checklist for items that should be reported in matched cohort studies.

The primary outcome is first psychiatric admission defined as requiring admission to a psychiatric unit (as recorded in Scottish Morbidity Record 04) after cancer diagnosis, compared using cumulative incidence functions with controls. The ICD-10 World Health Organisation system for classifying diseases, symptoms and causes of death was used to categorise cancer and psychiatric diagnoses. Four categories were studied: patients with all cancer diagnoses, central nervous system (CNS) diagnoses (any C7xx ICD10 diagnosis in the cancer registry data), lymphomas and leukaemias (any C8xx or C9xx ICD10 cancer registry diagnosis) and all cancer diagnoses other than CNS or lymphoma and leukaemia. Gray’s test for equality of cumulative incidence functions was used to estimate overall p values for differences in two cumulative incidence curves, again adjusted for the competing risk of death before first psychiatric admission. The Kalbfleisch-Prentice method was used for 95% CIs for incidence curves. Relative risks (RRs) of first psychiatric admission were calculated at 15 years and 25 years after diagnosis, with a RR less than one indicating a significant outcome from membership of the cancer survivor group, and with a 95% CI for the RR not including one indicating a statistically significant outcome.

We also investigated incidence rates per 1000 years follow-up for first admission and all admissions for nine categories of psychiatric admission: (1) all conditions—ICD10 codes Fxxx; (2) organic mental disorders—ICD10 codes F0xx; (3) substance use—ICD10 codes F1xx; (4) schizophrenia and related disorders—ICD10 codes F2xx; (5) mood disorders—ICD10 codes F3xx; (6) anxiety and stress-related disorders—ICD10 codes F4xx; (7) behavioural syndromes associated with physiological disturbances and physical factors—ICD10 codes F5xx; (8) personality disorders—ICD10 codes F6xx; (9) intellectual disability, neurodevelopmental disorders, other disorders arising in childhood—ICD10 codes F7xx, F8xx or F9xx. ICD code F99x (mental disorder not otherwise specified) was not present in the data; admissions for rehabilitation (ICD code Z50) or observation (ICD code Z03) were excluded from the study. As each psychiatric admission can be labelled with up to five ICD10 codes, we recorded an incidence event for every code that appears and hence the total number of incidence events is greater than the total number of admissions. Incidences were compared using a two-sided Poisson test for the null hypothesis that the incidence is the same for both groups, with significance set at 5%. We report incidence rate ratios (cancer survivor incidence divided by control group incidence) for first psychiatric admissions and rate ratios (cancer survivor rate of admission divided by control group rate of admission) for analyses involving all psychiatric admissions after cancer diagnosis. In both cases, a value less than one indicates a significant outcome for the cancer survivor group. Times to first admission were compared using a two-sided Wilcoxon rank-2 test with continuity correction for the null hypothesis that the time between diagnosis and first psychiatric admission is the same for both groups.

We also performed a post hoc subgroup analysis to investigate any relationship between childbirth (defined as having a live birth recorded in the Scottish Morbidity Record 02 registry) after cancer diagnosis and later psychiatric admission. Incidence rates per 1000 years follow-up for first and all psychiatric admissions were stratified by experience of any live birth for cancer survivors and controls. Those having a live birth during the study were further stratified into instances where the live birth preceded the first psychiatric admission or otherwise.

ICD-10 version: 2019^10^ was used to categorise all cancer and psychiatric admissions. Our data contained ICD9 codes which were converted to ICD10 using an online tool.^11^ All data linking, chart production and analysis were performed using R V.4.2.1. This analysis was approved by the NHS Scotland Public Benefit and Privacy Panel for Health and Social Care ref 1819–0186.

## Results

The cancer survivor group consisted of 3677 female children, teenagers and young adults (ages 0–24 years) diagnosed with cancer from 1981 to 2012 inclusive and followed up for a total of 60 740 years. Controls consisted of three matches for each cancer survivor case (n=11 031) followed up for 175 539 years. Mean follow-up was 16.5 years for both groups.

**Figure 1:**
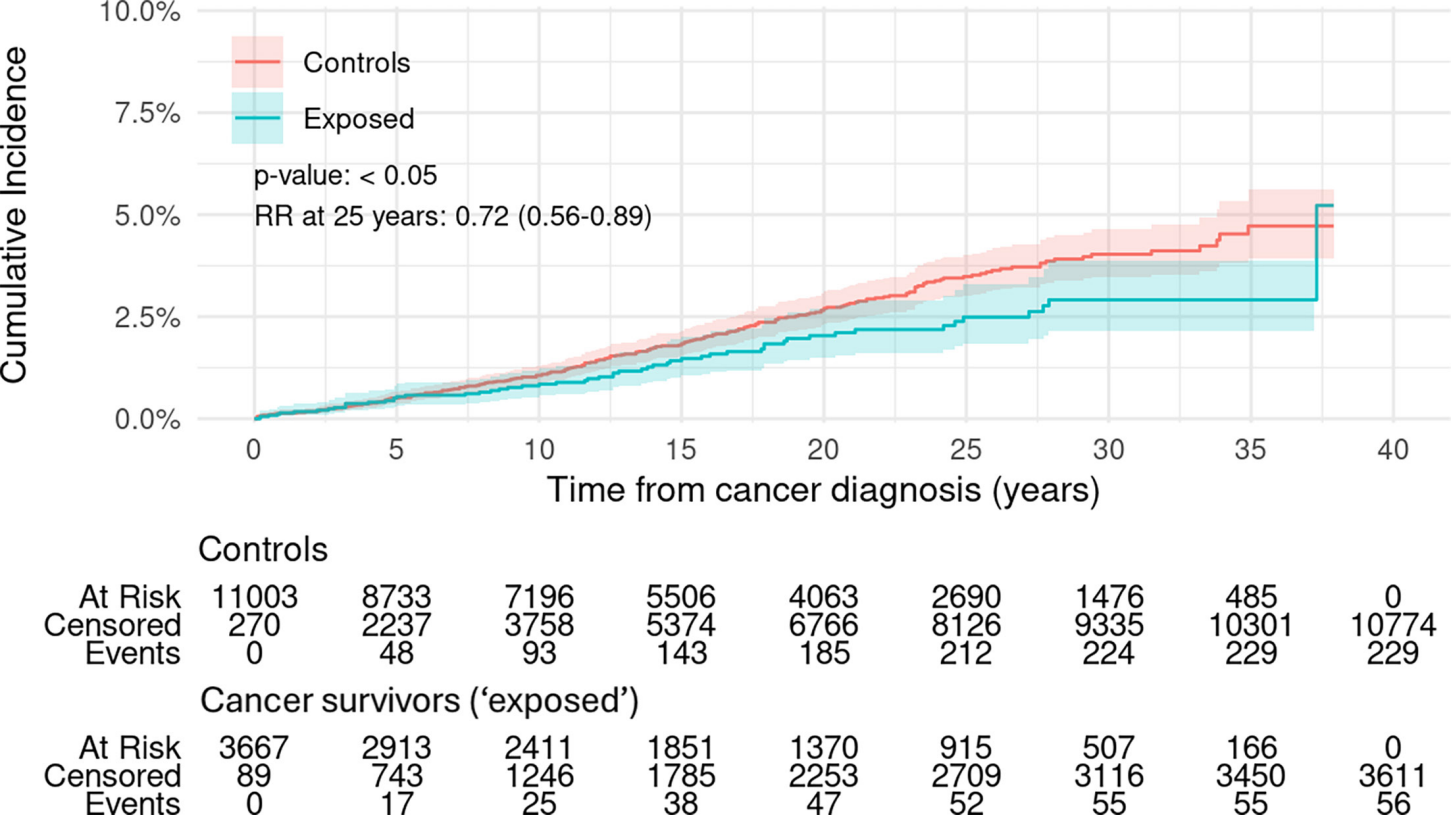
Cumulative incidence of first psychiatric admission in female cancer survivors aged 0–24 years at diagnosis for any cancer and matched controls. Overall p value is from Gray’s test for equality of cumulative incidence functions. Kalbfleisch-Prentice is used for 95% CIs for incidence curves. Events are first psychiatric admissions. Controls are adjusted for the competing risk of death for their exposed match. RR denotes the risk after 25 years follow-up of psychiatric admission in the cancer survivors relative to that in the control group, with values less than one favouring the cancer survivors. RR, relative risk.

Female cancer survivors had a significantly lower cumulative incidence of first psychiatric admissions than controls over the 38 years of follow-up for the cohort (p<0.05, figure 1). The RR of having a first psychiatric admission at 25 years from cancer diagnosis was 0.72 (0.56–0.89) (figure 1).

**Figure 2:**
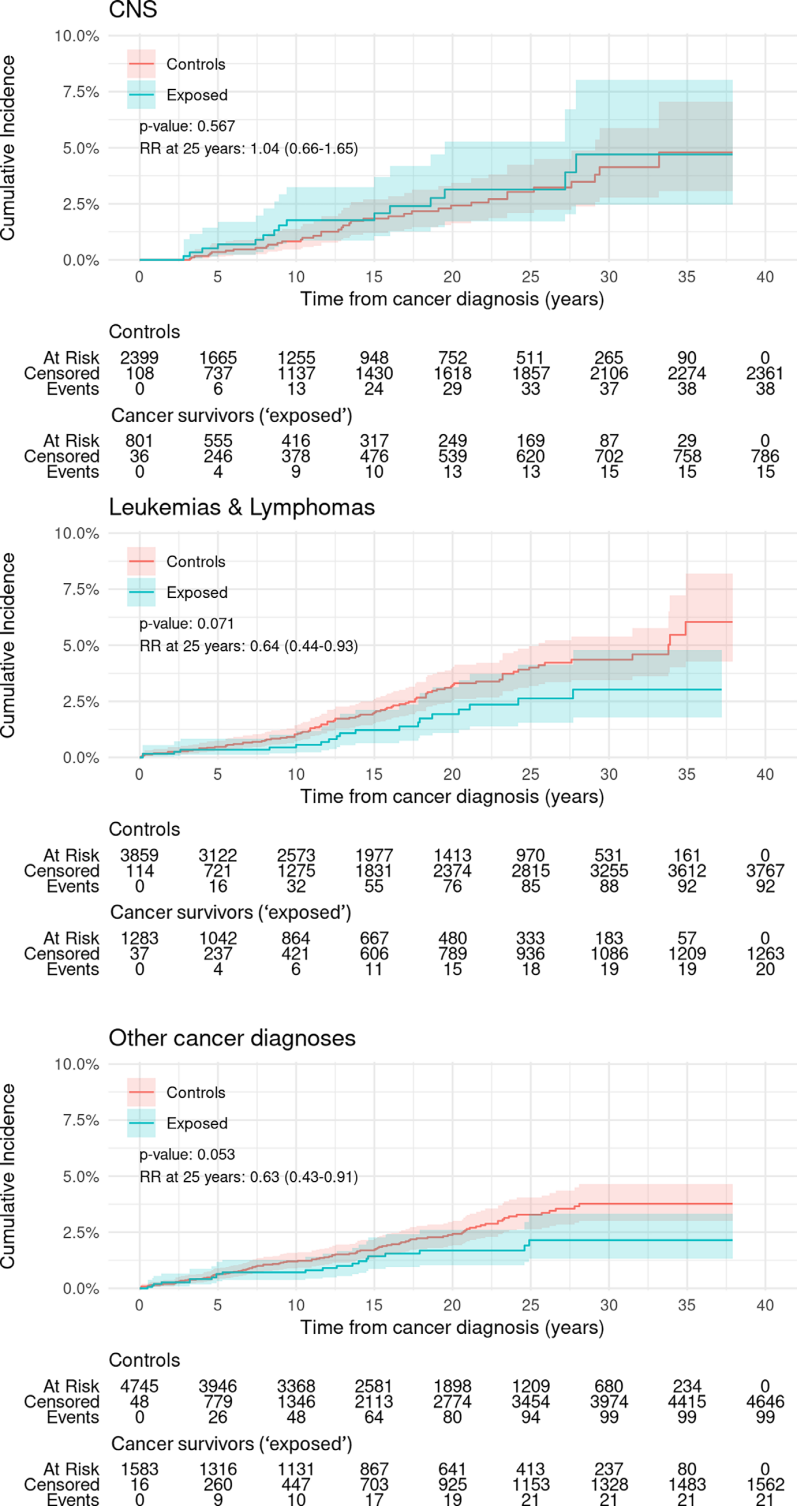
Cumulative incidence of first psychiatric admission in female cancer survivors aged 0–24 years at diagnosis and matched controls for CNS, leukaemia’s and lymphomas and other cancer types. Upper panel: cancers associated with the central nervous system (CNS). Centre panel: leukaemias and lymphomas. Lower panel: all other types of cancer. Overall p value is from Gray’s test for equality of cumulative incidence functions. Kalbfleisch-Prentice is used for 95% CIs for incidence curves. Events are first psychiatric admissions. Control group members are adjusted for the competing risk of death for their cancer survivor match. RR denotes the risk after 25 years follow-up of psychiatric admission in the cancer survivors relative to that in the control group, with values less than one favouring the cancer survivors. RR, relative risk.

Survivors of CNS cancer diagnoses had a similar cumulative incidence of first psychiatric admission to controls (figure 2 upper panel) with RR 1.04 (0.66–1.65) at 25 years from diagnosis. Survivors of lymphomas and leukaemias had lower but not statistically significant cumulative incidence of psychiatric admissions over the entire follow-up period than controls (p=0.071, figure 2 centre panel), as did survivors of cancers other than CNS, lymphomas and leukaemias (p=0.053, figure 2 lower panel). However, for both these two groups, the RR at 25 years indicated significant protection: for survivors of lymphomas and leukaemias, RR was 0.64 (0.44–0.93) and for survivors of other diagnoses, RR was 0.63 (0.43–0.91).

Survivors of childhood and young adult cancer had lower incidence per 1000 person years follow-up of a first psychiatric admission than controls (0.91 per 1000 person years for cancer survivors, 1.67 for controls, p<0.001) (table 1 upper panel). When stratified by ICD10 code group, there was a four-fold lower incidence of admissions for substance abuse (F1xx) (0.16 per 1000 years for cancer survivors, 0.61 for controls, p<0.001) and a two-fold lower incidence of mood disorders (F3xx) (0.48 vs 0.92, p<0.001) anxiety and stress-related disorders (F4xx) (0.26 vs 0.69, p<0.001) and personality disorders (F6xx) (0.18 vs 0.41, p<0.01 (table 1a). Only organic mental disorders (F0xx) had a higher incidence in cancer survivors than controls (0.08 vs 0.02, p<0.01) (table 1 upper panel). Rates of psychiatric admission for schizophrenia and related psychoses did not differ significantly between cancer survivors and controls. The differences in admission frequency were similar when counting all psychiatric admissions and not just the first such admission (table 1 lower panel).

**Table 1:** Incidence per 1000 person years follow-up for cancer survivors and matched control group. ICD code denotes the ICD-10 World Health Organisation classification system for diseases and symptoms.

ICD code	Description—first admission	N cancer survivors	N controls	Incidence cancer survivor	Incidence controls	Incidence rate ratio	P value
All F codes	All conditions	55	305	0.91	1.67	0.54	<0.001
F0xx	Organic mental disorders	*	*	0.08	0.02	4.00	<0.01
F1xx	Substance use	10	112	0.16	0.61	0.27	<0.001
F2xx	Schizophrenia and related disorders	14	49	0.23	0.27	0.86	NS
F3xx	Mood disorders	29	168	0.48	0.92	0.52	<0.001
F4xx	Anxiety and stress-related disorders	16	107	0.26	0.59	0.45	<0.001
F5xx	Behavioural syndromes associated with physiological disturbances and physical factors	5	17	0.08	0.09	0.88	NS
F6xx	Personality disorders	11	74	0.18	0.41	0.45	<0.01
F7xx, F8xx, F9xx	Intellectual disability, neurodevelopmental disorders, other disorders arising in childhood	7	15	0.12	0.08	1.40	NS

ICD code	Description—all admissions	N cancer survivors	N controls	Incidence cancer survivor	Incidence controls	Rate ratio	P value
All F codes	All conditions	195	1207	3.21	6.62	0.48	<0.001
F0xx	Organic mental disorders	*	*	0.12	0.02	7.00	<0.001
F1xx	Substance use	29	309	0.48	1.70	0.28	<0.001
F2xx	Schizophrenia and related disorders	41	165	0.68	0.91	0.75	NS
F3xx	Mood disorders	64	427	1.05	2.34	0.45	<0.001
F4xx	Anxiety and stress-related disorders	29	174	0.48	0.95	0.50	<0.001
F5xx	Behavioural syndromes associated with physiological disturbances and physical factors	9	38	0.15	0.21	0.71	NS
F6xx	Personality disorders	35	285	0.58	1.56	0.37	<0.001
F7xx, F8xx, F9xx	Intellectual disability, neurodevelopmental disorders, other disorders arising in childhood	32	75	0.53	0.41	1.28	NS

Upper panel: first psychiatric admission. Lower panel: all psychiatric admissions. The incidence rate ratio favours the cancer survivor group when less than one. An asterisk denotes a number too small to disclose safely.

* An asterisk denotes a number too small to disclose safely.

Median time to first psychiatric admission was not statistically significantly different between cancer survivors and controls for any category of psychiatric admission (10.0 years for cancer survivors and 11.7 years for controls) or for any of the four diagnosis categories: all diagnoses, CNS cancers, lymphomas and leukaemias or other diagnoses. There was no difference in the number of conditions recorded at psychiatric admission for the two groups: 100% had a main condition, 31% a second condition, 10% a third condition and 3% a fourth condition.

First live birth rates after cancer were different for the two groups, with no live birth in 67.9% of cancer survivors compared with 45.8% of controls. The median age at live birth was not significantly different between cancer survivors (26 years, IQR (22-30)) and controls (29 years (IQR 24–34), Mood test p>0.05). When nulliparous cancer survivors and controls are compared for first and all psychiatric admission, no significant difference in incidence per 1000 years follow-up is seen (table 2; and online supplemental tables 1a, b and 2a, b). However, when cancer survivors and controls who have experienced a live birth during the period of follow-up are compared, a positive outcome emerges for the cancer survivors (table 2 and online supplemental table 3). When the parous cases were subdivided into live birth followed by first psychiatric admission and vice versa, the increased incidence of psychiatric admission in controls was greatest for admissions after live births, but numbers were too small to make robust conclusions.

**Table 2:** Incidence per 1000 person years follow-up of first psychiatric admission stratified by parous and non-parous and further stratified for the parous cases into whether the first live birth preceded or followed the first psychiatric admission. ICD code denotes the ICD-10 World Health Organisation classification system for diseases and symptoms.

ICD code	Description	N cancer survivors	N controls	Incidence cancer survivor	Incidence controls	Incidence rate ratio	P value
All F codes	Nulliparous	27	63	0.44	0.35	1.29	NS
All F codes	Parous	28	242	0.46	1.33	0.35	<0.001
All F codes	First live birth followed by first psychiatric admission	18	168	0.30	0.92	0.32	<0.001
All F codes	First psychiatric admission followed by first live birth	10	74	0.16	0.41	0.41	<0.01

## Discussion

In this whole population Scottish linkage study, we have shown that young female cancer survivors are less likely to have a psychiatric admission after their cancer diagnosis than a matched control group. Admissions for mood disorders, anxiety and stress-related disorders, personality disorders and substance abuse are significantly less likely in the cancer survivors. The nature of the cancer diagnosis matters: survivors of leukaemia/lymphoma or other solid tumours are less likely to experience a psychiatric admission than controls, whereas survivors of CNS tumours have a similar risk of a psychiatric admission. Interestingly, having a first live birth is associated with an increased incidence of psychiatric admission in controls which is not seen in cancer survivors; this was the main driver for the overall reduction in psychiatric admission seen in female cancer survivors.

The published literature in this area is limited and largely at variance with our findings. Many mental health problems experienced by survivors of childhood cancer have been identified, but the limitations of the existing data, highlighted by systematic review, include uncertainty over their prevalence and risk factors.^7^ In a Danish nationwide, population-based, retrospective cohort study in 2003,^12^ the risk of hospitalisation for any psychiatric disease was higher among the survivors than in the general population, but the excess risk was restricted to survivors of brain tumour. The British Childhood Cancer Survivor Study^13^ also reported significantly higher levels of what was termed ‘mental health dysfunction’ than in the general population, with increases observed particularly among CNS and bone sarcoma survivors. Importantly, however, that study used a self-reported questionnaire for data collection and comparison with UK norms rather than matched individuals and is thus difficult to compare with the present analysis.

A more recent Scandinavian study^6^ including both male and female cancer survivors (<20 years at diagnosis) demonstrated the overall rates of incident psychiatric disorders during the study period were 34% higher among childhood cancer survivors than among the matched population (HR 1.34, 95% CI 1.28 to 1.39). The authors found a higher incidence among childhood cancer survivors than matched individuals for a majority of the 13 different groups of psychiatric disorders considered, with HRs for those with significant effects ranging from 1.19 (95% CI 1·02 to 1.39) for schizophrenia and other non-affective psychoses to 8·45 (5.52–12.93) for organic psychoses. Much of the increased risk overall was again driven by the survivors of CNS tumours, which we did not find, although that diagnostic group did not show the reduced incidence seen in the other types of cancer considered here. A key difference with our study is that the Scandinavian study recorded psychiatric hospital contacts (including outpatient and emergency contacts) rather than only admissions, so will include less severe disorders. This, at least in part, accounts for the much higher incidence of psychiatric interactions in that study versus psychiatric admissions here (5.81 per 1000 person years for childhood cancer survivors compared with 0.91 here and 4.43 for their matched controls vs 1.87 here). Additionally, both males and females were included in the Scandinavian study, with males making up 53.7% of the study population. Mood disorders are more prevalent in females than males^14^ and as the most common psychiatric diagnosis (ICD3xx) in our study population with a large difference in incidence between cancer survivors and the control group, this may also underlie some of the difference between these analyses. We also find differences by cancer diagnosis, particularly that survivors of brain cancers are not protected from mental disorders requiring psychiatric admission. Neurocognitive deficits after traumatic brain injury^15^ and after radiation therapy for brain cancers are well described^16^ and may account for this difference.

A study from St. Jude Children’s Research Hospital (SJCRH) examined the prevalence of emotional, behavioural and psychiatric outcomes in child and adolescent 5-year survivors of childhood acute lymphoblastic leukaemia (ALL) treated on a chemotherapy-only protocol between 2000–2010.^17^ Self-reported and parent-reported emotional and behavioural symptoms were assessed and compared with population expectations (10%), more survivors self-reported symptoms of inattention (27.9), hyperactivity/impulsivity (26.0%) and oppositional-defiant behaviour (20.1%). They concluded that a significant minority of survivors of ALL have long-term psychiatric morbidity.

The postpartum period is a time of greatly increased risk of psychiatric admission for females.^18^,^20^ As childhood cancer survivors are less likely to have children,^21 22^ we compared the rates of psychiatric admission in those who were nulliparous against those who had experienced a live birth. This is the first such study to make this comparison in cancer survivors. We found that there was no difference in rates of psychiatric admission in nulliparous cancer survivors compared with our control group, but that the large increase in risk of admission associated with childbirth seen as expected here in our control group was not seen in cancer survivors. Also, as expected, the increase in rates of psychiatric admission in controls occurred after childbirth, not before. Age at childbirth can influence the risk of psychiatric admission,^20^ but there was no significant difference in age at first live birth in our two groups.

The explanation for our findings remains speculative and will require further research. It is possible that cancer survivors and their families who have been heavily engaged with hospital services and should be part of a risk-stratified long-term follow-up programme have a different pattern of interaction with healthcare services. They may be more able to access support and interventions that may avoid psychiatric admission. It is also possible that female cancer survivors, particularly those without a CNS diagnosis, are more resilient to severe mental health disorders than non-exposed individuals for reasons which are unclear and require further research. When experiencing mental distress, childhood cancer survivors in Switzerland have been found to be more likely to access mental healthcare than their equally distressed siblings.^23^ It is also possible that cancer survivors and their social support networks are better equipped to manage psychiatric disorders without resorting to hospital admission, which is usually reserved for acute mental health crises when support in the community is unfeasible. The reasons for the different effects of childbirth on severe mental illness requiring admission in cancer survivors and our control group are unclear but could again relate to the potentially higher social support and engagement with services experienced by the cancer survivors, as the postpartum period is a particularly vulnerable and isolated time for many mothers.^24^ It is possible that with longer follow-up the perceived positive consequence for parous cancer survivors may be reduced.

The strengths of our study include that it is a whole population and thus not subject to selection bias, the lengthy follow-up period, the inclusion of a control group matched on key parameters, and the novel linkage to maternity records. The limitations of our study include that our whole population mental health registry is restricted to psychiatric admissions and does not capture other mental health outcomes such as diagnoses of severe mental illness managed only in the community or general hospital admissions following self-harm or complications of addiction or crisis attendances which do not result in admission. We acknowledge that we are not able to follow-up individuals who do not die but left Scotland. We estimate that this is a very small proportion of both the exposed and control groups, but may potentially be higher in controls. As the control group is drawn from the normal population, we have not accounted for the presence of marital status or lifetime behaviours or other diseases in our controls (beyond cancer) as potential confounders that could also potentially be associated with subsequent psychiatric admissions. Our study only includes female survivors of cancer who were nulliparous at cancer diagnosis, but due to the age distribution of the study population (under age 25 years at diagnosis), that is the great majority. Furthermore, despite being a 40-year whole population linkage study, the small numbers in some psychiatric diagnostic categories, cancer diagnoses and maternity events preclude robust interpretation of some sub-analyses due to lack of statistical power.

In conclusion, in this whole population Scottish linkage study, we have shown that young female cancer survivors are less likely to have a psychiatric admission after their cancer diagnosis than a matched control group. Admissions for mood disorders, anxiety and stress-related disorders, personality disorders and substance abuse are significantly less likely in the cancer survivors. This positive finding was restricted to those who also had a live birth, suggesting future studies should include parity in analyses. The explanations for our findings may relate to the experience of cancer treatment and survival shaping the personal characteristics or healthcare interactions of young people and their families in ways which protect against psychiatric admission in later life.

## Supplementary material

10.1136/bmjment-2025-301683 online supplemental file 1

## Data Availability

Data may be obtained from a third party and are not publicly available.
