# Supplementary material for: Psychiatric admission in female survivors of childhood and young adult cancer: a whole population retrospective study
Source: BMJ Ment Health. 2026 Feb 26;29(1):e301683. doi: 10.1136/bmjment-2025-301683 (PMC12958883; doi:10.1136/bmjment-2025-301683)
Supplement: online supplemental file 1 [file bmjment-29-1-s001.docx]

**Supplementary Information**

**Supplementary Table 1a. Incidence per 1,000 person years follow-up of first psychiatric admission for nulliparous cancer survivors and matched control group.**

| ICD code | Description | N Cancer survivors | N Controls | Incidence cancer survivors | Incidence controls | Incidence rate ratio | p value |
| --- | --- | --- | --- | --- | --- | --- | --- |
| All F codes | All conditions | 27 | 63 | 0.44 | 0.35 | 1.29 | NS |
| F1xx | Substance use | 5 | 13 | 0.08 | 0.07 | 1.15 | NS |
| F2xx | Schizophrenia and related disorders | 7 | 9 | 0.12 | 0.05 | 2.33 | <0.05 |
| F3xx | Mood disorders | 14 | 38 | 0.23 | 0.21 | 1.11 | NS |
| F4xx | Anxiety and stress-related disorders | 6 | 18 | 0.10 | 0.10 | 1.00 | NS |
| F5xx | Behavioural syndromes associated with physiological disturbances and physical factors | 0 | 4 | 0.00 | 0.02 | 0.00 | NA |
| Other F codes |  | 18 | 27 | 0.30 | 0.15 | 2.00 | <0.01 |

**Supplementary Table 1b. Incidence per 1,000 years follow-up of first psychiatric admission for parous cancer survivors and matched control group.**

| ICD code | Description | N Cancer survivors | N Controls | Incidence cancer survivors | Incidence controls | Incidence rate ratio | p value |
| --- | --- | --- | --- | --- | --- | --- | --- |
| All F codes | All conditions | 28 | 242 | 0.46 | 1.33 | 0.35 | <0.001 |
| F1xx | Substance use | 5 | 99 | 0.08 | 0.54 | 0.15 | <0.001 |
| F2xx | Schizophrenia and related disorders | 7 | 40 | 0.12 | 0.22 | 0.52 | NS |
| F3xx | Mood disorders | 15 | 130 | 0.25 | 0.71 | 0.35 | <0.001 |
| F4xx | Anxiety and stress-related disorders | 10 | 89 | 0.16 | 0.49 | 0.34 | <0.001 |
| F5xx | Behavioural syndromes associated with physiological disturbances and physical factors | 5 | 13 | 0.08 | 0.07 | 1.15 | NS |
| Other F codes |  | 5 | 65 | 0.08 | 0.36 | 0.23 | <0.001 |

**Supplementary Table 2a. Incidence per 1,000 years follow-up of all psychiatric admissions for nulliparous cancer survivors and matched control group.**

| ICD code | Description | N Cancer survivors | N Controls | Incidence cancer survivors | Incidence controls | Risk ratio | p value |
| --- | --- | --- | --- | --- | --- | --- | --- |
| All F codes | All conditions | 100 | 274 | 1.65 | 1.50 | 1.09 | NS |
| F1xx | Substance use | 18 | 25 | 0.30 | 0.14 | 2.16 | <0.01 |
| F2xx | Schizophrenia and related disorders | 14 | 15 | 0.23 | 0.08 | 2.80 | <0.001 |
| F3xx | Mood disorders | 30 | 103 | 0.49 | 0.57 | 0.87 | NS |
| F4xx | Anxiety and stress-related disorders | 14 | 33 | 0.23 | 0.18 | 1.27 | NS |
| F5xx | Behavioural syndromes associated with physiological disturbances and physical factors | 0 | 25 | 0.00 | 0.14 | 0.00 | NA |
| Other F codes |  | 64 | 157 | 1.05 | 0.86 | 1.22 | NS |

**Supplementary Table 2b. Incidence per 1,000 years follow-up of all psychiatric admissions for parous cancer survivors and matched control group.**

| ICD code | Description | N Cancer survivors | N Controls | Incidence cancer survivors | Incidence controls | Risk ratio | p value |
| --- | --- | --- | --- | --- | --- | --- | --- |
| All F codes | All conditions | 95 | 933 | 1.56 | 5.12 | 0.31 | <0.001 |
| F1xx | Substance use | 11 | 284 | 0.18 | 1.56 | 0.12 | <0.001 |
| F2xx | Schizophrenia and related disorders | 27 | 150 | 0.44 | 0.82 | 0.54 | <0.001 |
| F3xx | Mood disorders | 34 | 324 | 0.56 | 1.78 | 0.31 | <0.001 |
| F4xx | Anxiety and stress-related disorders | 15 | 141 | 0.25 | 0.77 | 0.32 | <0.001 |
| F5xx | Behavioural syndromes associated with physiological disturbances and physical factors | 9 | 13 | 0.15 | 0.07 | 2.08 | <0.05 |
| Other F codes |  | 10 | 206 | 0.16 | 1.13 | 0.15 | <0.001 |

**Supplementary Table 3. Incidence per 1,000 years follow-up of first psychiatric admission for cancer survivors and matched control group that had a maternity event, stratified by mood disorder and whether or not the first maternity event preceded the first psychiatric admission (PA).**

|  | Maternity then PA | | | | |
| --- | --- | --- | --- | --- | --- |
|  |  |  |  |  |  |
| ICD code | N Cancer survivors | N Controls | Incidence cancer survivors | Incidence controls | p value |
| All F codes | 18 | 168 | 0.30 | 0.92 | <0.001 |
| F3xx | 9 | 86 | 0.15 | 0.47 | <0.001 |
| Other F codes | 23 | 263 | 0.38 | 1.44 | <0.001 |
|  |  |  |  |  |  |
| 0 | PA then Maternity | | | | |
|  |  |  |  |  |  |
| ICD code | N Cancer survivors | N Controls | Incidence cancer survivors | Incidence controls | p value |
| All F codes | 10 | 74 | 0.16 | 0.41 | <0.01 |
| F3xx | 6 | 44 | 0.10 | 0.24 | <0.05 |
| Other F codes | 9 | 83 | 0.15 | 0.46 | <0.001 |
